# Effect of progestin-based contraceptives on HIV-associated vaginal immune biomarkers and microbiome in adolescent girls

**DOI:** 10.1371/journal.pone.0306237

**Published:** 2024-07-15

**Authors:** Mélodie A. Nasr, Annette Aldous, Jason Daniels, Christopher Joy, Eleanor Capozzi, Michelle Yang, Patricia Moriarty, Vanessa Emmanuel-Baker, Sharyn Malcolm, Stefan J. Green, Veronica Gomez-Lobo, Mimi Ghosh

**Affiliations:** 1 Department of Epidemiology, George Washington University, Washington, DC, United States of America; 2 Department of Biostatistics and Bioinformatics, George Washington University, Washington, DC, United States of America; 3 MedStar Washington Hospital Center, Washington, DC, United States of America; 4 Children’s National Hospital, Washington, DC, United States of America; 5 Genomics and Microbiome Core Facility, Rush University, Chicago, IL, United States of America; 6 National Institute of Child Health and Human Development, National Institutes of Health, Washington, DC, United States of America; Mount Sinai School of Medicine: Icahn School of Medicine at Mount Sinai, UNITED STATES OF AMERICA

## Abstract

Adolescent girls bear a disproportionate burden of both the HIV epidemic and unintended pregnancies; yet important questions remain unanswered regarding the effects of hormonal contraceptives on the vaginal immune microenvironment, which can impact HIV susceptibility in this group. Multiple studies report genital immune alterations associated with the progestin-based contraceptive Depot medroxyprogesterone acetate (DMPA) in adult women, but there is little available data in adolescents. The objective of this longitudinal cohort study was to evaluate the effects of short-term use of three progestin-based contraceptives, levonorgestrel intrauterine device (LNG-IUD), subdermal etonogestrel (ETNG), and injectable DMPA, on HIV-associated vaginal immune biomarkers and microbiome in adolescent girls. Fifty-nine sexually active, HIV-uninfected girls aged 15-19, were recruited from the Washington DC metro area and self-selected into Control (condoms only), combined oral contraceptive pills, LNG-IUD, ETNG and DMPA groups. Vaginal swabs were collected at baseline prior to contraceptive use and at 3-month follow-up visit. Vaginal secretions were tested for pro-inflammatory (IL-1α, IL-1β, TNF-α, IL-6, IL-8, MIP-3α, IP-10, RANTES, MIP-1α, MIP-1β) and anti-inflammatory/anti-HIV (Serpin-A1, Elafin, Beta-Defensin-2, SLPI) immune biomarkers using ELISA and for anti-HIV activity using TZM-bl assay. Vaginal microbiome was evaluated using 16S rRNA gene sequencing. Data were analyzed using SAS Version 9. Among the 34 participants who completed both visits, no significant changes in median biomarker concentrations, HIV inhibition and microbiome composition were observed between baseline and follow-up visits for any of the contraceptive groups. IL-8 (p<0.01), MIP-3α (0.02), Elafin (p = 0.03) and RANTES (p<0.01) differed significantly by race whereas IL-6 was significantly different by age (p = 0.03). We conclude that 3-month use of LNG-IUD, ETNG and DMPA have minimal effects on adolescent vaginal immune microenvironment, and therefore unlikely to impact HIV risk. Future studies with larger sample size and longer follow-up are recommended to continue to evaluate effects of contraceptives on the lower genital tract immunity and susceptibility to sexually transmitted infections.

## Introduction

Adolescent girls and young women bear a disproportionate burden of sexually transmitted infections (STI) including HIV, accounting for 63% of new infections in the Sub-Saharan Africa in 2021 [1]. This population also experiences high rates of often-unplanned pregnancies and needs access to safe and effective contraceptives. However, adolescents have been severely under-represented in studies focusing on contraceptives and HIV risk [2, 3].The few existing studies that do include adolescents have demonstrated a distinct physiological and immunological microenvironment in adolescent genital tract, emphasizing the need for evaluation of contraceptive use and risk of HIV/STI acquisition in this population [4–9].

Adolescent girls have an immature cervix (cervical ectopy), characterized by a thinner epithelium that is easier to breach than the mature adult cervix, thus facilitating pathogen entry [4, 5]. Ectopy has been linked to increased risk of STI, including HIV [8], and is influenced by a variety of factors including age, hormonal levels, oral contraceptive usage, sexual debut, number of sexual partners and local inflammation [5, 9–13]. Adolescent genital tract can also have higher baseline inflammation [4, 6] and dysbiotic microbiome [7] compared to adult women, which can increase susceptibility to HIV.

Immune responses in the female genital tract (FGT) are intricately regulated by sex hormones, estrogen and progesterone [14]. Investigation of biological mechanisms of HIV susceptibility in women have demonstrated that both endogenous and exogenous sex hormones can affect infectivity and pathogenesis [15, 16]. Ex-vivo and animal studies have reported enhanced HIV infection in the luteal phase of the menstrual cycle when levels of the sex hormone progesterone is high [17, 18].

Depot Medroxyprogesterone Acetate (DMPA), an injectable progestin-based contraceptive, is highly effective in preventing pregnancies and is a popular choice in Sub-Saharan Africa where adolescent girls are particularly susceptible to HIV acquisition. Although earlier studies reported an increased risk of HIV acquisition in adult women using DMPA [19–21] more recent studies did not share these findings [3, 22]. However, changes in genital immune microenvironment following DMPA usage in adult women have been well documented. Specifically, multiple studies have reported thinning of the vaginal epithelium, dysregulated cellular and soluble immune mediators and a dysbiotic microbiome [23–31]. In contrast, majority of studies have not found detrimental effects of other injectable progestin-based contraceptives such as norethisterone enanthate (NET-N) [3]. Other progestin-based contraceptives include levonorgestrel intrauterine device (LNG-IUD) and subdermal etonogestrel (ETNG) implants, which are the more popular choices among girls in the USA. A handful of studies have reported activation of inflammatory pathways and reduced expression of tight junction proteins in genital tract epithelial cells following use of LNG-IUD [3, 24, 32–35]. However, no association with increased risk of HIV acquisition has been reported. Data regarding progestin-based subdermal implants (LNG and ETNG) in this regard has been sparse and although no increased HIV risk has been identified [3, 35], a recent study in adult women found slightly increased FGT HIV target cells in ETNG users [36].

Currently there are major knowledge gaps regarding our understanding of adolescent FGT immune responses and the effects of progestin-based contraceptives including DMPA. Additionally, most studies, including the single randomized clinical trial (ECHO) [3], were conducted in global settings and little information is available about adolescents in the USA. With this in mind, our objective in this study was to longitudinally evaluate short-term effects of three progestin-based contraceptives on vaginal immune biomarkers and microbiome in a cohort of adolescent girls in the Washington DC metro area.

## Materials and methods

### Study design and approval

This is a longitudinal cohort study, which enrolled sexually active HIV negative adolescent girls aged 15-19 years, from Medstar Washington Hospital Center and the Adolescent Clinic at Children’s National Hospital in Washington DC between 8.30.2017 to 8.1.2019. The Institutional Review Boards (IRB) at Children’s National and MedStar Washington Hospital Center and George Washington University approved the study protocol at each site. Patients were approached upon presentation for contraceptive counseling and informed about the study. Interested patients were screened and consent or assent was obtained from those eligible and willing to participate. Because the adolescents were accessing care for confidential services (namely contraception), waiver of parental consent was granted by all IRBs. Parental consent waiver was applied for all cases. Per IRB requirements, adolescents consented if they were aged 18+ and assented if 12-17 years old.

Participants self-selected into treatment arms: (1) control group (no prescribed contraceptive, condom only), (2) combined oral contraceptives pill (OCP), (3) levonorgestrel (LNG) intrauterine device (IUD), (4) etonogestrel implant (ETNG) and (5) DMPA injectable. Only those who were assigned female sex at birth, not received puberty suppression or cross sex hormones and underwent menarche at least 1 year before study enrollment were included. Participants were excluded if they were known to be HIV-infected, had abnormal vaginal discharge indicative of genital infection at the time of visit, using copper IUD, pregnant/breast-feeding, on hormonal contraceptives, had engaged in vaginal sex or used lubricant products 48 hours prior to study procedures and/or taken immunomodulatory medications for the past 3 months. The study staff had access to identifiable participant information, however all biological samples were deidentified prior to analysis and storage. One follow-up visit was conducted 3 months after contraceptive initiation and behavioral data and biological samples were collected at both visits.

### Demographic Information and bio-specimen collection

Participants responded to a survey that included questions about medical history, substance abuse, smoking habits, sexual behavior and gynecologic and reproductive history at baseline and follow-up. At each visit, providers collected vaginal secretions non-invasively (without use of a speculum) by inserting a sterile Dacron swab about 2 inches into the vagina and gently swabbing the lateral vaginal walls. A second swab was similarly collected for microbiome analysis and a third swab was collected for testing of Chlamydia, Gonorrhea, and Trichomonas. If symptomatic, testing for Candida and Bacterial vaginosis (BV) was also conducted. If lab results indicated presence of an infection, the sample was excluded and biospecimen collection was rescheduled following treatment.

### Specimen processing

Swabs were placed in 1 ml 1X PBS (pH 7) and transported to the research lab within 3 hours of collection. Swabs were vortexed briefly and compressed against the side of the tube to maximize elution of secretions. The eluent was clarified twice at 700×g for 10 minutes, aliquoted, and stored at −80°C. Swabs collected for microbiome analysis were placed dry in a cryovial, stored at -80°C and batch shipped to University of Illinois at Chicago for DNA-based 16S ribosomal RNA (rRNA) gene amplicon sequencing.

### Detection of immune biomarkers

Vaginal secretions were assayed for cytokines: TNF-α (DY210), IL-6 (DY206), IL-1α (DY200), IL-1β (DY201); chemokines: MIP-3α (DY360), IL-8 (DY208), IP-10 (DY266), MIP-1α (DY270), MIP-1β (DY271), RANTES (DY278); antimicrobial/anti-HIV: secretory leukocyte protease inhibitor (SLPI, DP100), Elafin (DY1747), Human beta defensin 2 (HBD2), Serpin A1, using Quantikine or Duoset ELISA kits. All kits except HBD-2 and Serpin A1 were purchased from R&D Systems (Minneapolis, MN). HBD2 (900-K172) and Serpin A1 (LS-F4915) ELISA kits were obtained from PeproTech (Rocky Hill, NJ) and Lifespan Biosciences (Seattle, Washington), respectively. Biomarkers were quantified based on standard curves obtained using a Microplate Reader (Biotek, Winooski, VT). Biomarker concentrations below the lower limit of detection were reported as the mid-point between the lowest concentrations measured and zero. Total protein concentration was determined using the Pierce BCA protein assay kit (Thermo Fisher Scientific, Waltham, MA).

### Measurement of anti-HIV activity

HIV-1 BaL was kindly provided by Dr. P. Gupta (University of Pittsburgh, PA). Anti-HIV activity in vaginal secretions was determined using TZM-bl indicator cell-line (NIH HIV Reagent Program, Division of AIDS, NIAID, NIH)) as described previously [37, 38]. HIV inhibition was tested against HIV-1 BaL at 250 tissue culture infectious dose (TCID _50_). Viability was determined using the CellTiter 96 AQueous One Solution Cell Proliferation Assay (Promega, Madison, WI).

### Microbiome analysis

DNA extraction, library preparation, sequencing and data processing and annotation were performed at the Genome Research Core and the Research Informatics Core at the University of Illinois at Chicago. Genomic DNA (gDNA) was extracted from vaginal swabs using DNA extraction performed on a Qiagen EZ1 instrument, implementing the EZ1 DNA tissue protocol (Qiagen, Hilden, Germany). Genomic DNA from vaginal swabs was PCR amplified and prepared for next-generation sequencing using a modified two-step targeted amplicon sequencing approach, as described previously [39, 40]. Briefly, gDNA was used as template for an initial amplification with primers 515F and 806R containing Fluidigm common sequences (CS1 and CS2), targeting the V4 variable region of microbial 16S rRNA genes. A second PCR amplification was performed using primers obtained from the Access Array Barcode Library for Illumina sequencers (Fluidigm, South San Francisco, CA; Item# 100–4876), which contained Illumina sequencing adapters, a sample-specific barcode (reverse primer), and CS1 or CS2 sequences. Barcoded amplicons were pooled and sequenced on an Illumina MiniSeq sequencer, using a mid-output flow cell and 2x150 base sequencing.

Paired-end reads were merged using the software package PEAR v.0.9.6 [41] and trimmed using cutadapt v1.18 to remove ambiguous nucleotides and primer sequences [42]. Reads lacking the primer sequences and/or sequences shorter than 225 bases following merging and quality trimming (Q20) were discarded. Chimeric sequences were identified and removed using the USEARCH algorithm with a comparison to Silva v132 reference sequence [43, 44]. Amplicon sequence variants were identified using DADA2 v1.18 [45] and annotated taxonomically using the Naïve Bayesian classifier [46] included in DADA2 with the SILVA v132 training set.

### Power calculation

The study was designed as a pilot, with the aim of recruiting approximately 50 participants, n = 10 for each of the 5 groups. This would give us good statistical power only for relatively strong effects. For example, a one-sample t-test would have 80% power to detect a moderately large standardized effect size of 1.0.

### Statistical analysis

Demographic characteristics were compared using Fisher’s Exact Test. Biomarkers were log-10 transformed and evaluated as continuous variables. Biomarker measures were compared between groups using Wilcoxon rank sum, Kruskal-Wallis, and Dunn’s with Bonferroni adjustment tests. Biomarkers were analyzed both with and without normalization to total protein content, and the two methods yielded similar results, with no differences seen in the statistical significance of the resulting associations. We report the results of the non-normalized analysis. For microbiome analyses, OTU abundance counts were transformed to proportions. Shannon diversity and proportion of Lactobacillus (genus, species *iners*, species non-*iners*) were compared between subgroups using Kruskal-Wallis test and between visit (overall and by contraceptive group) with Wilcoxon rank-sum test. Differences in community structure from baseline to follow-up visit were assessed using Bray-Curtis dissimilarity and compared between contraceptive groups with Kruskal-Wallis test. Spearman correlation coefficients were calculated for biomarker heat-map analysis, excluding biomarkers detected in fewer than 35% of samples. Missing data were minimal (0 for most variables, 1-2 for MIP-1α, MIP-1β, and SLPI measurements, and 5 for SerpinA1). We noted these in table footnotes and excluded cases with missing data from significance tests involving the missing variables. Data from participants who were lost to follow-up after visit 1 were included in the baseline analyses but excluded from the paired analyses. All analyses were conducted with SAS 9.4 and R 4.2. Microbiome analyses used R packages phyloseq and vegan.

## Results

### Characteristics of study population at baseline and follow-up

A total of 59 participants completed the baseline visit: 64% were Black, 22% White and 14% were of other or mixed race; 61%were 15-17 years old, and 39% were 18-19 years old. The majority of participants experienced their first sexual encounter between the ages of 15-17 years (75%), never drank alcohol (61%), and never smoked cigarettes or marijuana (90%). The prevalence of current Candida (9%) or BV (0%) was low, and the majority of participants had 1-4 total lifetime sexual partners (83%) (Table 1).

**Table 1:** Characteristics of study participants.

	All Samples	Paired Samples: Baseline and Follow-Up, N = 34^a^					
	All Baseline	Control^b^	OCP	LNG	ETNG	DMPA	*P* Value^c^
**N**	59	6	7	9	7	5	
**Age Group**							
** 15-17**	36 (61.0)	1 (16.7)	6 (85.7)	9 (100.0)	1 (14.3)	2 (40.0)	**<0.001**
** 18-19**	23 (39.0)	5 (83.3)	1 (14.3)	0 (0.0)	6 (85.7)	3 (60.0)	
**Race**							
** Black**	38 (64.4)	6 (100.0)	3 (42.9)	2 (22.2)	5 (71.4)	5 (100.0)	**0.003**
** White**	13 (22.0)	0 (0.0)	2 (28.6)	7 (77.8)	1 (14.3)	0 (0.0)	
** Other**	8 (13.6)	0 (0.0)	2 (28.6)	0 (0.0)	1 (14.3)	0 (0.0)	
**Regular Cycles**							
** Yes**	45 (76.3)	6 (100.0)	4 (57.1)	5 (55.6)	7 (100.0)	4 (80.0)	0.10
** No**	14 (23.7)	0 (0.0)	3 (42.9)	4 (44.4)	0 (0.0)	1 (20.0)	
**Age at First Sex**							
** 12-14**	9 (15.3)	2 (33.3)	0 (0.0)	1 (11.1)	1 (14.3)	1 (20.0)	0.27
** 15-17**	44 (74.6)	3 (50.0)	7 (100.0)	8 (88.9)	4 (57.1)	4 (80.0)	
** 18-19**	6 (10.2)	1 (16.7)	0 (0.0)	0 (0.0)	2 (28.6)	0 (0.0)	
**Lifetime Partners**							
** 1**	30 (50.8)	0 (0.0)	4 (57.1)	7 (77.8)	1 (14.3)	3 (60.0)	**0.04**
** 2-4**	19 (32.2)	4 (66.7)	2 (28.6)	1 (11.1)	5 (71.4)	2 (40.0)	
** 5+**	10 (16.9)	2 (33.3)	1 (14.3)	1 (11.1)	1 (14.3)	0 (0.0)	
**Alcohol Use**							
** Never**	36 (61.0)	5 (83.3)	3 (42.9)	5 (55.6)	3 (42.9)	3 (60.0)	0.71
** 1-3 drinks per month**	16 (27.1)	1 (16.7)	3 (42.9)	1 (11.1)	3 (42.9)	1 (20.0)	
** 1+ drinks per week**	7 (11.9)	0 (0.0)	1 (14.3)	3 (33.3)	1 (14.3)	1 (20.0)	
**Smoking**							
** Never**	53 (89.8)	6 (100.0)	7 (100.0)	9 (100.0)	4 (57.1)	5 (100.0)	0.06
** Past**	1 (1.7)	0 (0.0)	0 (0.0)	0 (0.0)	1 (14.3)	0 (0.0)	
** Current**	5 (8.5)	0 (0.0)	0 (0.0)	0 (0.0)	2 (28.6)	0 (0.0)	
**History of STI**							
** Never**	51 (86.4)	3 (50.0)	6 (85.7)	9 (100.0)	6 (85.7)	4 (80.0)	0.15
** Ever**	8 (13.6)	3 (50.0)	1 (14.3)	0 (0.0)	1 (14.3)	1 (20.0)	
**Current yeast infection**							
** Yes**	5 (8.5)	0 (0.0)	0 (0.0)	2 (22.2)	0 (0.0)	0 (0.0)	0.36
** No**	54 (91.5)	6 (100.0)	7 (100.0)	7 (77.8)	7 (100.0)	5 (100.0)	
**Current BV**							NA
** No**	59 (100.0)	6 (100.0)	7 (100.0)	9 (100.0)	7 (100.0)	5 (100.0)	

^a^Of 59 participants at baseline 34 returned for a follow-up visit, resulting in 34 paired samples.

^b^Control group used no prescribed contraceptives and was designated as “condom only”.

^c^Fisher’s Exact test comparing all five contraceptive groups at baseline, using samples from participants who completed two visits (N = 34).

*P* values < .05 shown in **bold**. NA: *p* value could not be calculated.

A follow-up visit was completed by 34 participants. In one case, no sample was collected because the participant was menstruating, and 5 samples were excluded due to presence of blood (n = 2), positive test for Chlamydia (n = 2), or self-treatment with Monostat (n = 1). In each case, the participant returned for a third visit and provided a usable sample. Of the 34 participants, 6 opted for no contraceptive (condom only), 7 selected OCP, 9 selected LNG IUD, 7 selected ETNG implant, and 5 selected injectable DMPA. Contraceptive choice differed significantly by age (*p* < 0.001), with younger participants preferring OCP or LNG-IUD and older participant choosing ETNG, DMPA, or no contraceptive. Contraceptive choice also differed significantly by race (*p* = 0.003) and the number of lifetime sexual partners (*p* = 0.04) (Table 1).

### Vaginal immune biomarkers at baseline (all participants)

**Fig 1:**
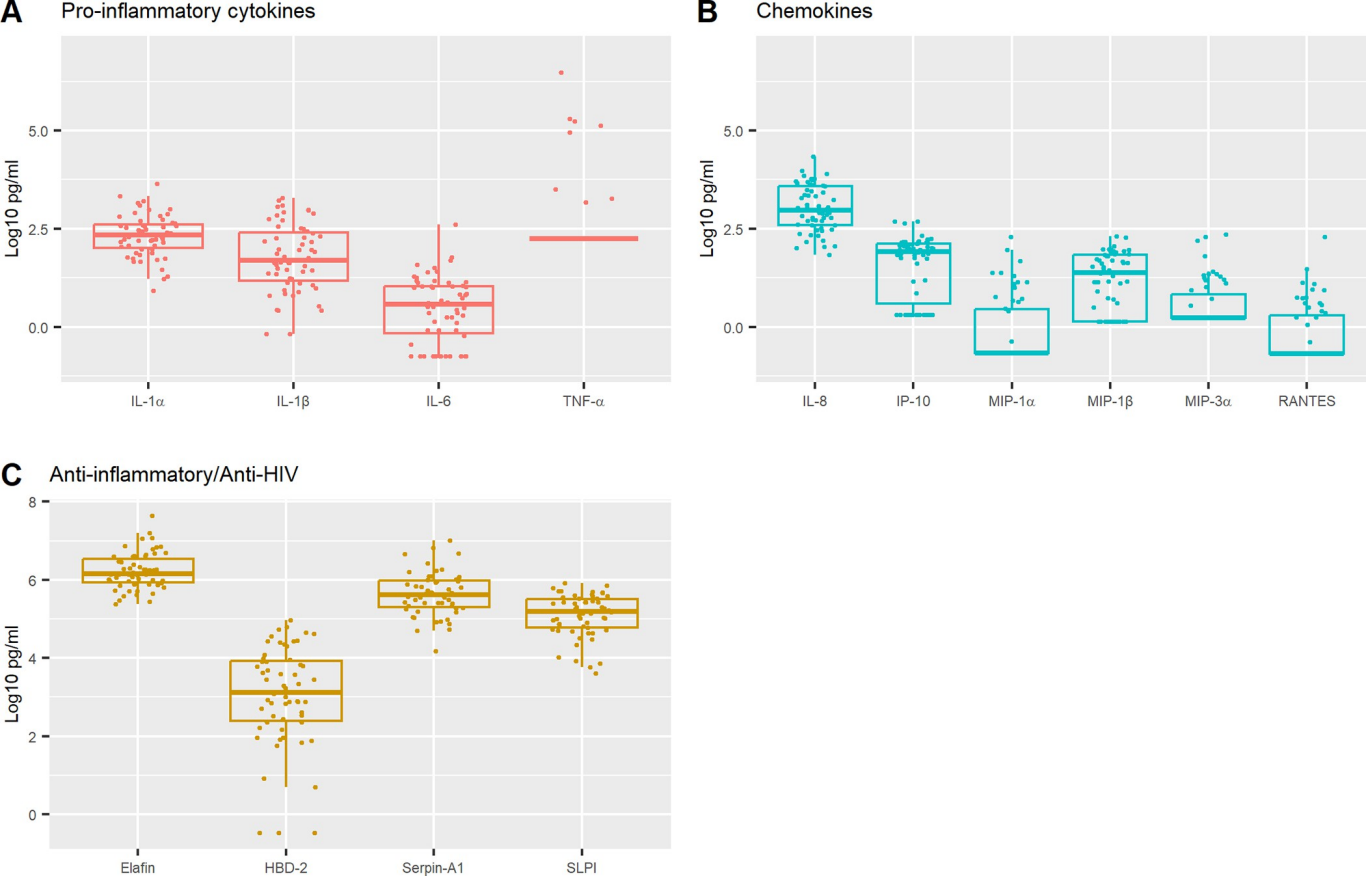
Vaginal Immune biomarkers in adolescent girls, at baseline. Vaginal fluids at baseline visit (no contraceptives) from adolescent girls (n = 59) were tested for **(A)** pro-inflammatory cytokines (IL-1α, IL-1β, IL-6, TNF-α), **(B)** chemokines (IL-8, IP-10, MIP-1α, MIP-1β, MIP-3α, RANTES), **(C)** anti-inflammatory/anti-HIV (Elafin, HBD-2, Serpin-A1, SLPI) by standard ELISA assays. Biomarker levels are shown in log 10 pg/mL and bars depict median.

Analysis of the distribution patterns of cytokines, chemokines and antimicrobial/anti-HIV biomarkers in the vaginal secretions of all participants at baseline was performed (Fig 1 and Table 2). Of the inflammatory cytokines/chemokines, we observed >90% detectability for IL-1α (median log 2.34 pg/ml), IL-1β (median log 1.69 pg/ml), and IL-8 (median log 2.97 pg/ml). IL-6 (median log 0.59 pg/ml) and IP-10 (median log 1.92 pg.ml) were detectable in 70—80% of samples. Fewer than 35% of samples had detectable levels of TNF-α (median log 2.25 pg/ml), MIP-1α (median log -0.66 pg/ml), MIP-3α (median log 0.24 pg/ml) and RANTES (median log -0.68 pg/ml). Mucosal antimicrobials with anti-inflammatory and anti-HIV functions, Elafin (median log 6.16 pg/ml), HBD-2 (median log 3.12 pg/ml), SerpinA1 (median log 5.62 pg/ml) and SLPI (median log 5.19 pg/ml) were detectable in 95-100% of the samples (Table 2).

**Table 2:** Concentrations of biomarkers at baseline by race and age group.

Marker^a^	Percent Detectable	All	Black	White	Other	*P* Value Race^c^	15-17	18-19	*P* Value Age^c^
**N**	59	59	38	13	8		36	23	
**IL-1α**	100	2.34 (2.01–2.61)	2.24 (1.91–2.65)	2.39 (2.23–2.55)	2.40 (2.22–2.62)	0.77	2.39 (2.16–2.61)	2.25 (1.93–2.59)	0.28
**IL-1β**	97	1.69 (1.17–2.41)	1.63 (1.02–2.34)	2.11 (1.41–2.71)	1.77 (1.34–2.28)	0.45	1.64 (1.20–2.33)	1.76 (1.24–2.46)	0.59
**IL-6**	78	0.59 (-0.15–1.04)	0.40 (-0.38–1.03)	0.74 (0.48–1.28)	0.70 (0.09–1.13)	0.25	0.65 (0.29–1.14)	-0.08 (-0.74–1.01)	**0.03**
**TNF-α**	14	2.25 (2.25–2.25)	2.25 (2.25–2.25)	2.25 (2.25–4.95)	2.25 (2.25–2.25)	0.08	2.25 (2.25–2.25)	2.25 (2.25–2.25)	0.09
**IL-8**	100	2.97 (2.60–3.59)	2.85 (2.49–3.32)	3.61 (3.11–3.77)	2.97 (2.70–3.64)	**0.007**	2.95 (2.60–3.59)	3.04 (2.66–3.53)	0.99
**IP-10**	75	1.92 (0.59–2.12)	1.94 (0.31–2.11)	1.90 (1.83–2.11)	1.89 (1.61–2.12)	0.77	1.85 (0.73–2.08)	1.99 (0.74–2.15)	0.3
**MIP-1α**	31	-0.66 (-0.66–0.45)	-0.66 (-0.66–0.66)	-0.66 (-0.66–1.14)	-0.66 (-0.66–0.51)	0.43	-0.66 (-0.66–0.65)	-0.66 (-0.66–0.66)	0.47
**MIP-1β**	73	1.38 (0.14–1.83)	1.52 (1.14–1.84)	0.14 (0.14–1.80)	0.42 (0.14–1.73)	0.09	1.15 (0.14–1.84)	1.51 (0.96–1.68)	0.55
**MIP-3α**	29	0.24 (0.24–0.84)	0.24 (0.24–0.24)	0.24 (0.24–1.01)	0.84 (0.47–1.32)	**0.02**	0.24 (0.24–1.14)	0.24 (0.24–0.24)	0.14
**RANTES**	32	-0.68 (-0.68–0.30)	-0.68 (-0.68–0.68)	-0.68 (-0.68–0.36)	0.75 (0.01–0.99)	**0.007**	-0.68 (-0.68–0.61)	-0.68 (-0.68–0.65)	0.08
**Elafin**	100	6.16 (5.94–6.53)	6.27 (6.01–6.61)	5.88 (5.72–6.15)	6.17 (6.10–6.31)	**0.03**	6.11 (5.87–6.41)	6.18 (6.13–6.63)	0.21
**HBD-2**	95	3.12 (2.39–3.93)	3.19 (2.38–4.39)	2.89 (2.22–3.58)	3.48 (2.88–3.95)	0.42	2.91 (1.96–3.83)	3.45 (2.78–3.95)	0.11
**Serpin-A1**	100	5.62 (5.30–5.99)	5.55 (5.18–5.83)	5.94 (5.62–6.08)	5.43 (5.26–6.01)	0.15	5.57 (5.27–6.00)	5.70 (5.40–5.96)	0.76
**SLPI**	100	5.19 (4.78–5.50)	5.18 (4.78–5.46)	5.01 (4.80–5.42)	5.34 (5.03–5.64)	0.55	5.24 (4.90–5.53)	5.08 (4.69–5.43)	0.31
**HIV Inhibition** ^**b**^		22.5 (0.65–35.3)	17.7 (-1.0–31.9)	35.3 (22.5–45.1)	17.3 (-0.3–34.9)	0.09	27.0 (-1.3–37.5)	11.1 (1.1–29.9)	0.34

^a^ % detectable and median (IQR) level, log_10_ pg/mL; For “All”, SerpinA1 data missing for 5 participants, SLPI, MIP-1α and MIP-1β missing for 1 participant.

^b^ Median (IQR) % inhibition.

^c^ *P* values < .05 shown in **bold** text, Kruskal-Wallis test.

Median baseline level of IL-8 differed significantly by participant’s race (*p* <0.01)), with highest level for White participants. Elafin was significantly lower among White participants (*p* = 0.03), while MIP-3α was highest in participants of other or mixed race (*p* = 0.02). HIV inhibition was higher among White participants (35% vs. 17-18%), but the difference was not statistically significant (*p* = 0.09). IL-6 was significantly higher among 14-17 year-olds compared to the older adolescents (*p* = 0.03) (Table 2).

### Vaginal immune biomarkers at baseline and follow-up (paired samples)

**Fig 2:**
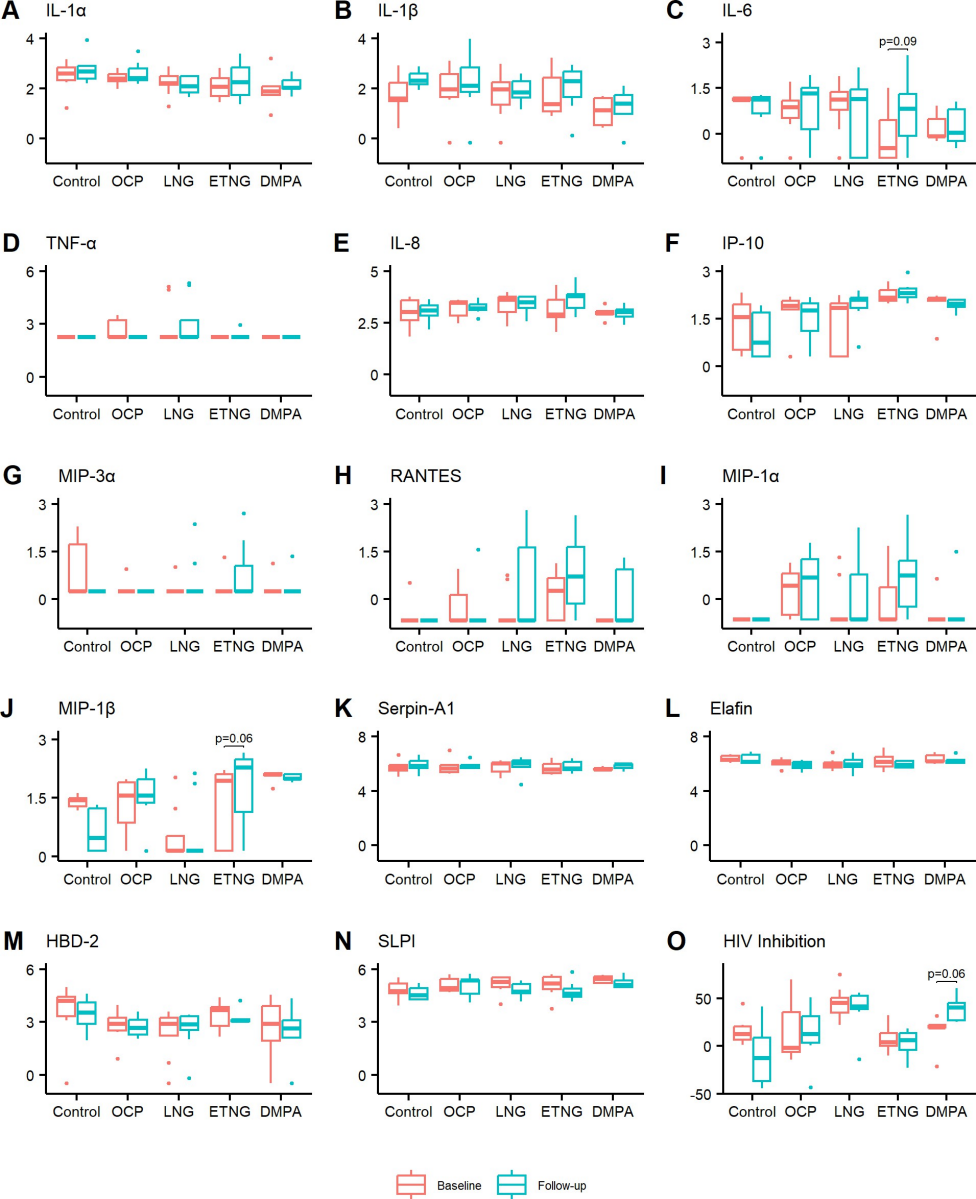
Vaginal immune biomarkers in adolescent girls at baseline and after 3 months of contraceptive use. Paired comparisons of vaginal immune biomarker levels were conducted for baseline (no contraceptive, red bars) vs follow-up (contraceptive, blue bars) visits in adolescent girls (n = 34). Biomarkers were measured by standard ELISA assays and included pro-inflammatory cytokines **(A-D)**, IL-1α, IL-1β, IL-6, TNF-α), chemokines **(E-J)**, IL-8, IP-10, MIP-3α, RANTES, MIP-1α, MIP-1β), and anti-inflammatory/anti-HIV biomarkers **(K-N)**, Serpin-A1, Elafin, HBD-2, SLPI). Anti-HIV activity in vaginal fluids is shown as %HIV inhibition **(O)**. Biomarker levels are shown in log 10 pg/mL and bars depict median.

Longitudinal analysis with paired tests was conducted comparing baseline and follow-up visits by contraceptive group. We found no statistically significant differences between visits in levels of cytokines (IL-1α, IL-1β, TNF-α, IL-6), chemokines (IL-8, MIP-3α, IP-10, RANTES, MIP-1α, MIP-1β), or antimicrobial/anti-HIV (Serpin-A1, Elafin, HBD2, SLPI) biomarkers, although MIP-1β (*p* = .06) and IL-6 (*p* = .09) trended toward higher levels at follow-up in the ETNG group (Table 3 and Fig 2). A sensitivity analysis using protein-normalized values produced similar results, with no statistically significant differences in biomarker levels between visits.

**Table 3:** Concentrations of biomarkers at baseline and 3-month follow-up, N = 34^a^.

Marker^b^	Group	Percent Detectable	Baseline Median (IQR)	Follow-Up Median (IQR)	Difference of Medians	*P* Value^c^
**IL-1α**	Control	100	2.60 (2.33-2.84)	2.69 (2.38-2.90)	0.08	0.44
	OCP	100	2.40 (2.28-2.58)	2.41 (2.34-2.80)	0.01	0.58
	LNG	100	2.21 (2.16-2.49)	2.08 (1.85-2.49)	-0.13	0.30
	ETNG	100	2.08 (1.69-2.42)	2.26 (1.74-2.84)	0.18	0.38
	DMPA	100	1.89 (1.74-2.08)	2.04 (1.99-2.33)	0.15	0.62
**IL-1β**	Control	100	1.60 (1.49-2.23)	2.31 (2.18-2.59)	0.70	0.16
	OCP	86	1.96 (1.66-2.58)	2.11 (1.86-2.85)	0.14	0.14
	LNG	94	1.97 (1.35-2.26)	1.85 (1.63-2.29)	-0.12	0.50
	ETNG	100	1.36 (1.08-2.44)	2.29 (1.66-2.67)	0.93	0.69
	DMPA	90	1.12 (0.54-1.63)	1.39 (0.99-1.75)	0.27	0.62
**IL-6**	Control	83	1.03 (1.02-1.11)	1.04 (0.62-1.13)	0.00	>.99
	OCP	86	0.81 (0.48-1.02)	1.23 (0.14-1.40)	0.43	0.40
	LNG	78	1.04 (0.74-1.28)	1.06 (-0.74-1.36)	0.02	0.73
	ETNG	64	-0.44 (-0.74-0.42)	0.76 (-0.07-1.22)	1.20	0.09
	DMPA	100	-0.08 (-0.08-0.44)	0.03 (-0.23-0.74)	0.11	>.99
**TNF-α**	Control	0	2.25 (2.25-2.25)	2.25 (2.25-2.25)	0.00	NA
	OCP	21	2.25 (2.25-3.22)	2.25 (2.25-2.25)	0.00	0.18
	LNG	28	2.25 (2.25-2.25)	2.25 (2.25-3.21)	0.00	0.18
	ETNG	7	2.25 (2.25-2.25)	2.25 (2.25-2.25)	0.00	>.99
	DMPA	0	2.25 (2.25-2.25)	2.25 (2.25-2.25)	0.00	NA
**IL-8**	Control	100	3.03 (2.63-3.58)	3.10 (2.84-3.34)	0.08	>.99
	OCP	100	3.46 (2.85-3.53)	3.20 (3.12-3.39)	-0.26	0.94
	LNG	100	3.61 (3.03-3.77)	3.48 (3.21-3.76)	-0.13	0.73
	ETNG	100	2.89 (2.78-3.61)	3.79 (3.21-3.87)	0.91	0.22
	DMPA	100	2.94 (2.91-3.08)	3.01 (2.81-3.14)	0.08	>.99
**IP-10**	Control	58	1.54 (0.52-1.96)	0.74 (0.31-1.69)	-0.81	0.10
	OCP	86	1.90 (1.79-2.06)	1.76 (1.11-1.98)	-0.15	0.58
	LNG	83	1.83 (0.31-1.99)	2.10 (1.83-2.16)	0.26	0.11
	ETNG	100	2.16 (2.07-2.40)	2.30 (2.17-2.45)	0.14	0.67
	DMPA	100	2.13 (2.07-2.16)	1.96 (1.86-2.10)	-0.16	0.86
**MIP-1α**	Control	8	-0.66 (-0.66--0.66)	-0.66 (-0.66--0.66)	0.00	NA
	OCP	64	0.41 (-0.51-0.80)	0.67 (-0.66-1.26)	0.26	0.79
	LNG	33	-0.66 (-0.66--0.66)	-0.66 (-0.66-0.77)	0.00	0.36
	ETNG	50	-0.66 (-0.66-0.36)	0.73 (-0.25-1.20)	1.39	0.10
	DMPA	20	-0.66 (-0.66--0.66)	-0.66 (-0.66--0.66)	0.00	>.99
**MIP-1β**	Control	83	1.34 (1.19-1.39)	0.44 (0.14-1.15)	-0.90	0.10
	OCP	86	1.45 (0.81-1.77)	1.45 (1.28-1.84)	0.00	0.53
	LNG	28	0.14 (0.14-0.50)	0.14 (0.14-0.14)	0.00	>.99
	ETNG	64	1.81 (0.14-1.96)	2.12 (1.06-2.32)	0.31	0.06
	DMPA	100	1.96 (1.94-1.99)	1.86 (1.85-1.97)	-0.10	>.99
**MIP-3α**	Control	17	0.24 (0.24-1.71)	0.24 (0.24-0.24)	0.00	0.37
	OCP	7	0.24 (0.24-0.24)	0.24 (0.24-0.24)	0.00	>.99
	LNG	17	0.24 (0.24-0.24)	0.24 (0.24-0.24)	0.00	0.42
	ETNG	21	0.24 (0.24-0.24)	0.24 (0.24-1.04)	0.00	0.42
	DMPA	20	0.24 (0.24-0.24)	0.24 (0.24-0.24)	0.00	>.99
**RANTES**	Control	8	-0.68 (-0.68--0.68)	-0.68 (-0.68--0.68)	0.00	>.99
	OCP	21	-0.68 (-0.68-0.13)	-0.68 (-0.68--0.68)	0.00	>.99
	LNG	33	-0.68 (-0.68--0.68)	-0.68 (-0.68-1.63)	0.00	0.20
	ETNG	64	0.24 (-0.68-0.65)	0.71 (-0.15-1.64)	0.46	0.11
	DMPA	20	-0.68 (-0.68--0.68)	-0.68 (-0.68-0.93)	0.00	0.37
**Elafin**	Control	100	6.28 (6.19-6.58)	6.11 (6.04-6.64)	-0.17	0.84
	OCP	100	6.14 (5.93-6.22)	5.91 (5.67-6.14)	-0.23	0.38
	LNG	100	5.92 (5.73-6.07)	5.93 (5.74-6.29)	0.02	0.82
	ETNG	100	6.12 (5.80-6.49)	5.88 (5.72-6.19)	-0.24	0.47
	DMPA	100	6.15 (6.15-6.60)	6.13 (6.06-6.27)	-0.01	>.99
**HBD-2**	Control	92	4.21 (3.31-4.44)	3.52 (2.88-4.10)	-0.69	0.44
	OCP	100	2.89 (2.52-3.23)	2.67 (2.28-3.12)	-0.23	0.81
	LNG	94	2.88 (2.22-3.23)	2.85 (2.55-3.33)	-0.02	>.99
	ETNG	100	3.68 (2.78-3.85)	3.06 (3.04-3.16)	-0.62	0.81
	DMPA	80	2.88 (1.92-3.91)	2.64 (2.12-3.10)	-0.25	0.81
**Serpin-A1**	Control	100	5.70 (5.47-5.91)	5.84 (5.69-6.20)	0.14	0.84
	OCP	100	5.65 (5.37-5.91)	5.84 (5.68-5.90)	0.19	0.56
	LNG	100	6.01 (5.41-6.10)	6.02 (5.73-6.23)	0.02	0.46
	ETNG	100	5.57 (5.31-5.99)	5.62 (5.54-6.14)	0.05	0.30
	DMPA	100	5.55 (5.55-5.67)	5.92 (5.69-5.94)	0.37	0.19
**SLPI**	Control	100	4.75 (4.63-5.19)	4.51 (4.28-4.92)	-0.24	0.69
	OCP	100	4.91 (4.79-5.46)	5.34 (4.61-5.37)	0.43	0.69
	LNG	100	5.27 (4.97-5.52)	4.73 (4.63-5.14)	-0.54	0.13
	ETNG	100	5.18 (4.86-5.56)	4.61 (4.42-4.89)	-0.57	0.22
	DMPA	100	5.46 (5.22-5.59)	5.11 (4.99-5.36)	-0.35	0.31
**Protein**	Control	100	2.95 (2.83-2.98)	2.96 (2.87-3.01)	0.00	0.69
	OCP	100	2.90 (2.87-2.98)	2.94 (2.90-3.02)	0.04	0.11
	LNG	100	2.92 (2.81-3.06)	2.89 (2.84-3.13)	-0.02	0.57
	ETNG	100	2.99 (2.89-3.02)	3.07 (3.03-3.13)	0.08	**0.03**
	DMPA	100	2.87 (2.57-2.93)	2.99 (2.75-3.08)	0.13	0.12
**HIV Inhibition** ^d^	Control	100	12.64 (6.80-20.85)	-12.69 (-36.97-8.98)	-25.33	0.19
	OCP	100	-1.71 (-6.19-35.76)	12.60 (3.53-31.52)	14.31	0.94
	LNG	100	45.06 (35.25-50.43)	41.76 (38.86-52.90)	-3.30	0.57
	ETNG	100	4.05 (0.18-13.73)	6.04 (-4.10-13.55)	1.99	0.47
	DMPA	100	19.79 (19.47-22.29)	40.26 (27.03-45.09)	20.48	0.06

^a^ Paired samples: n = 6 Control, 7 OCP, 9 LNG, 7 ETNG, 5 DMPA. SerpinA1 data missing for 2 participants (1 OCP, 1 LNG). MIP-1α and MIP-1β missing for 1 Control participant.

^b^ % detectable and median (IQR) level, log_10_ pg/mL.

^c^ *P* values < .05 shown in **bold** text, Wilcoxon Signed Rank test. NA: *p* value could not be calculated because at least one group had no detectable samples.

^d^ Median (IQR) % inhibition

### Anti-HIV activity at baseline and follow-up (paired samples)

As vaginal secretions are known to have innate HIV inhibitory activity [47, 48], we tested samples from baseline and follow-up visits using TZM-bl indicator cell line. Although no statistically significant differences were observed when comparing baseline and follow-up for each group, a trend toward higher HIV inhibition was seen in the DMPA group after 3 months of usage (40% vs 20%, *p* = .06) (Table 3 and Fig 2).

### Vaginal microbiome analysis at baseline and follow-up (paired samples)

Vaginal microbiome is an integral component of female genital immunity and can be modulated by endogenous and exogenous sex hormones [23, 49]. We classified the vaginal microbiomes in our samples according to the “cervicotypes” (CTs), described by Anahtar et al. [50]. Twenty one of our samples belonged to CT1 (dominated by non-*iners Lactobacillus*); 29 to CT2 (dominated by *L*.*iners*); 9 to CT3 (high relative abundance of *Gardnerella)*; and 9 to CT4 (diverse community, including *Prevotella* species). Bacteria from the genera *Atopobium*, *Sneathia* and *Magasphaera* were common in the CT3 and CT4 groups. Samples from Black participants (64.4% of all participants) were spread across all four CTs with CT2 being the most common for this group (45%, 19/42), though most individuals with CT3 and CT4 microbiomes were Black (78%, 7/9 in each). CT1 was the most common among White participants (60%, 12/20) followed by CT2 (30%, 6/20). S1 Fig shows analysis of vaginal microbiome in this cohort by race, visit and contraceptive group.

**Fig 3:**
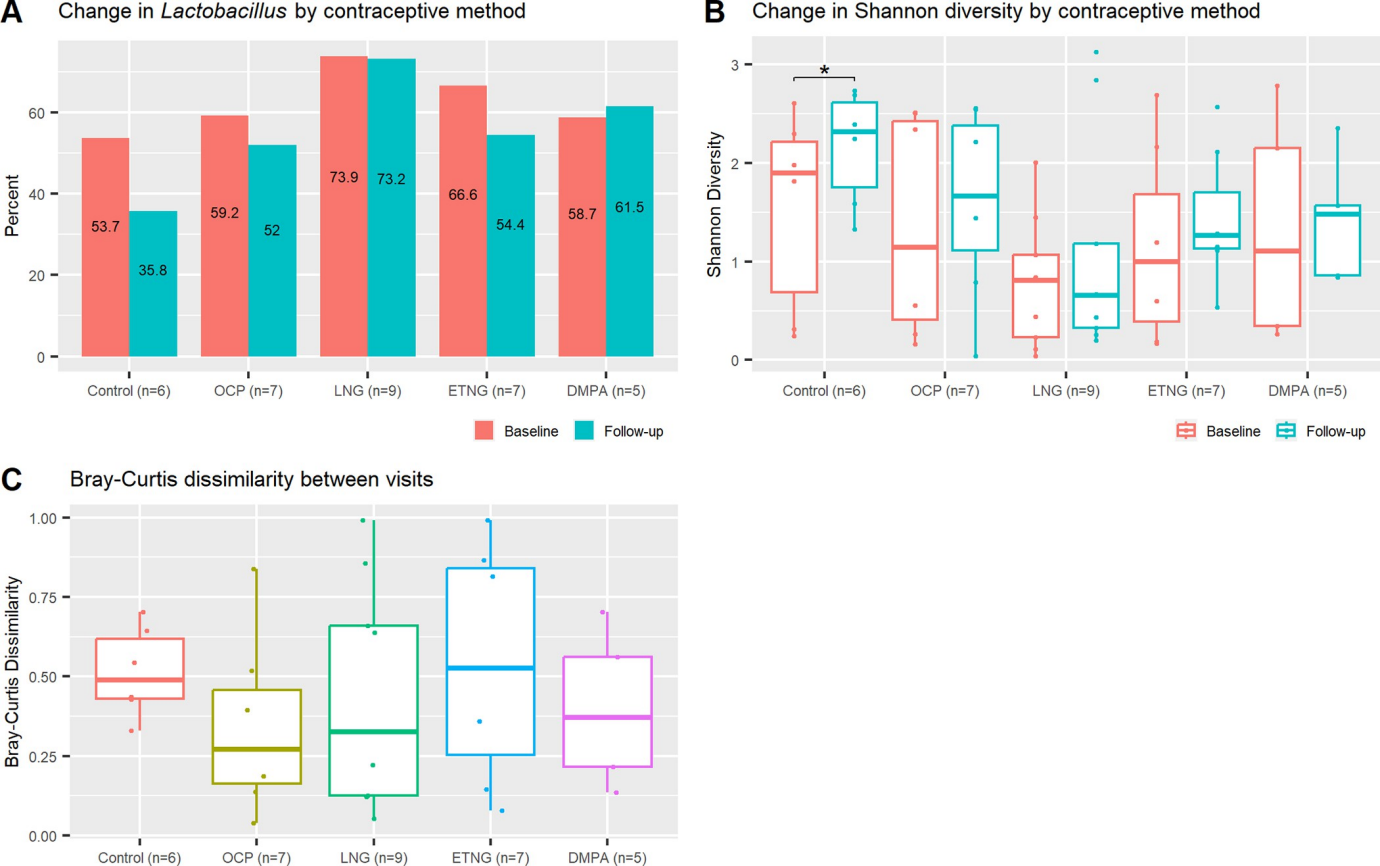
Vaginal microbiome in adolescent girls at baseline and after 3 months of contraceptive use. Vaginal microbiome composition was analyzed in paired samples (n = 34) by contraceptive group. A) Change in % *Lactobacillus* from baseline to follow-up; B) change in Shannon alpha-diversity from baseline to follow-up; and C) Bray-Curtis dissimilarity between baseline and follow-up.

The mean relative abundance of *Lactobacillus* ranged from 54-74% in all contraceptive groups at baseline and from 36-73% at follow-up. The lowest relative abundance was observed in the Control group and the highest in the LNG-IUD group at both time points with no significant changes observed from baseline to follow-up (Fig 3A). When stratified by species, there were no significant differences from baseline to follow-up for *L*.*iners* or other species within the genus *Lactobacillus*.

Alpha diversity (Shannon index) was not significantly different between groups at baseline (*p* = .57) or follow-up (*p* = .16), although diversity in the LNG-IUD group was notably lower than in the Control group at both time points. Longitudinal paired analysis showed no significant changes in Shannon diversity from baseline to follow-up except, unexpectedly, in the Control group (*p* = .03) (Fig 3B). Bray-Curtis distance from baseline to follow-up was greatest in the ETNG group (median .53) and least in the OCP (.27) group, but the difference between the five groups was not statistically significant (*p* = .69) (Fig 3C).

### Heat-map analysis of all immune variables at baseline and follow-up

**Fig 4:**
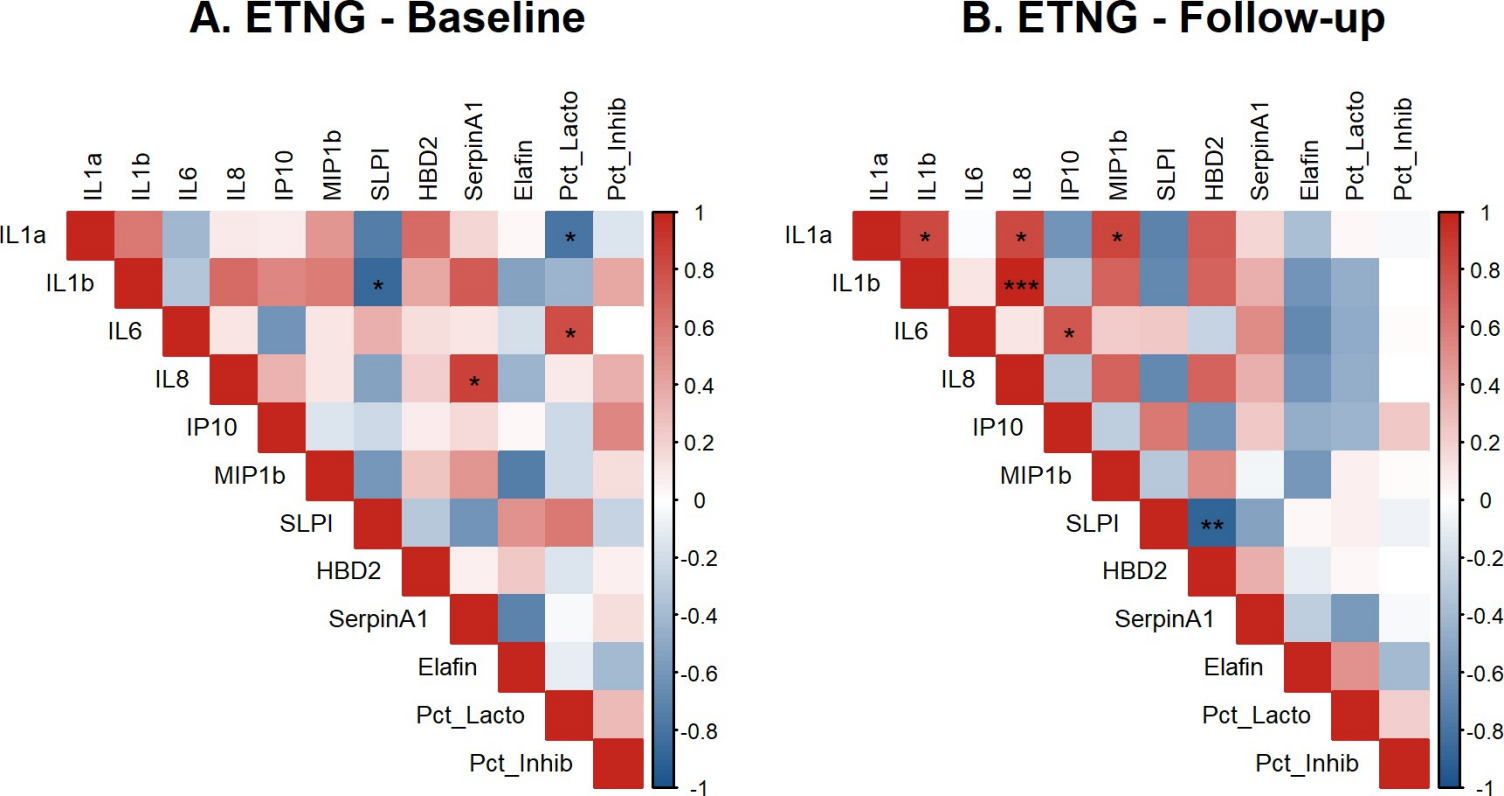
Correlation between immune biomarkers and HIV inhibition in ETNG group at baseline and follow-up. Heat map of Spearman’s coefficients show significant changes in ETNG contraceptive group comparing baseline and follow-up visits. Each cell of the heat map denotes the Spearman correlation coefficients. Cells highlighted in shades of RED indicate a positive association with darker shades representing stronger associations. Similarly, cells highlighted in shades of BLUE indicate a negative association with darker shades representing stronger associations. Statistically significant (*p* < .05) associations are denoted with *. Missing numbers (white) indicate there was no variation within that subgroup usually due to undetectable values, therefore, the correlation coefficient could not be calculated. Biomarkers with levels below the limit of detection for >90% of samples were excluded from this analysis.

Distinct patterns of synergistic or antagonistic clustering of HIV associated immune biomarkers by hormonal status or sexual activity have been previously noted [6, 7, 51, 52]. Heat-map analyses of correlations among immune variables revealed no significant clustering differences in baseline vs follow-up for the OCP, LNG-IUD and DMPA groups. We observed significant clustering of inflammatory biomarkers IL-1α, IL-1β, IL-8 and MIP-1β, in the follow-up visit for ETNG, which was absent at the baseline visit (Fig 4).

## Discussion

We report comprehensive characterization of soluble immune biomarkers and microbiome in the vaginal compartment before and after short-term use of progestin-based contraceptives in adolescent girls in the USA. We observed no significant changes in vaginal immune biomarkers and microbiome following 3-month usage of progestin-based contraceptives LNG-IUD, ETNG and DMPA. However, we did find significant associations between race and age with specific immune biomarkers. We also report here, baseline genital immune biomarker distribution patterns in healthy USA adolescent girls aged 15-19, and add to the literature where other populations have been similarly evaluated [9, 53, 54].

Multiple previous studies have reported genital immune dysregulation in adult women using DMPA. DMPA usage was also linked to increased HIV acquisition, although more recent studies have not confirmed these findings [3, 22]. Our findings from this study contribute to the literature by focusing on adolescent girls, a population where such data is lacking.

Although we did not observe statistically significant changes in vaginal biomarkers for any of the contraceptive groups, we did note a trend toward increased anti-HIV activity (p = 0.06) among the DMPA users. Previous studies in a cohort of adult women have reported suppression of multiple genital immune biomarkers along with a decrease in anti-HIV activity in cervical-vaginal lavage (CVL) after 3 months of DMPA use [27]. It is possible that our findings differ due to the younger age range of our cohort. It is also possible that the increased anti-HIV activity may also be due to activity of other unknown proteins that were not measured in our panel. Proteomic studies have identified >100 distinct proteins in the FGT and many remain uncharacterized to date [55–57].

An interesting observation was the significantly high levels of IL-8 and reduced levels of Elafin in White participants along with higher levels of HIV inhibition. A possible mechanism to explain this may be the involvement of neutrophils, since IL-8 chemo-attracts neutrophils and Elafin modulates neutrophil activity by inhibiting neutrophil elastase and proteinase 3 [58]. Neutrophil mediated immune protection against HIV in the female genital tract has been reported [59] although their activity in the vagina in the context of contraceptives remains unclear. Additionally, White race has been associated with higher neutrophil counts compared to non-White individuals [60].

The uniqueness of our adolescent cohort and the method of sample collection and assays that were used may also explain why the concentrations of biomarkers we report are higher than other publications. We opted to use non-invasive vaginal swabs whereas several other studies have used menstrual cups or CVL, which can result in distinct biomarker profiles [6, 7, 9, 27, 49, 53, 54, 61–63]. Additionally, whereas we used ELISA, others have used multiplex assays [9, 49, 61], which can result in differences in absolute values of biomarkers [63–65]. Genital immune biomarker profile can also vary based on cervical ectopy [4, 63, 66].

An interesting finding was our observation of higher concentration of total protein (p = 0.03), inflammatory cytokine IL-6 (p = 0.09) and chemokine MIP1-β (p = 0.06) following ETNG use, along with distinct clustering among inflammatory biomarkers. To our knowledge, this has not been reported previously, although, a recent study in adult women found minimal increase in FGT HIV target cells among ETNG users [36]. This same study also described slight reductions in soluble immune biomarkers in CVL, which is in contrast to our findings in adolescent girls.

Vaginal microbiome composition has been repeatedly shown to impact HIV risk with a *Lactobacillus crispatus* dominant microbiome associated with protection against HIV/STI acquisition [67–69], and a polymicrobial microbiome (BV), associated with elevated risk [9, 69–71]. We observed a relatively stable, Lactobacilli predominant microbiome in our cohort that did not significantly change following contraceptive use. It is also interesting that our cohort had no diagnosed symptomatic BV and low prevalence of Candida, Chlamydia, Gonorrhea, and Trichomonas, although the rates of STI in adolescent girls are considered to be on the rise [72]. Vaginal microbiome is also known to be associated with race/ethnicity with Black and Hispanic women having a baseline non-Lactobacilli predominant microbiome while showing no BV type pathology [67]. Although our cohort consisted of 64% Black adolescents, we did not observe significant differences in relative abundance of Lactobacilli in any group. The LNG-IUD group, with majority White participants, had lower microbial diversity. This may be because this group had the highest relative abundance of bacteria from the genus *Lactobacillus* to begin with. Dysbiotic vaginal microbiome has also been reported following DMPA use in Black and Hispanic but not White women [73]. Although not included in our study, non-hormonal IUD, such as copper IUD, has been shown to result in dysbiotic vaginal microbiome and increased inflammatory immune profile in multiple studies [35, 74, 75].

As in previous studies we noted differences in contraceptive choice based on age and race [76], with a higher proportion of Black adolescents selecting injectable contraception and younger adolescents selecting OCP and IUD. Patients were cared for by adolescent medicine and pediatric gynecology providers in different settings (hospital vs Ob/Gyn clinic) which may have influenced contraception selection [77]. We observed an association of IL-8, MIP-3α and RANTES with race and IL-6 with age. Such associations have been previously described [7, 53, 54] and likely affected by menstrual regularity, ectopy, and sexual activity [5, 6, 61, 63].

A major strength of our study is the analyses of baseline immune biomarkers in 59 adolescent girls from the USA. To the best of our knowledge, this is the largest study conducted among girls in the USA focusing on DMPA and HIV-associated biomarkers. Baseline immune profiling of adolescent FGT in the context of HIV/STI risk, has been primarily conducted in girls from Sub-Saharan Africa [9, 49, 53] and Europe [54, 61], with only four studies in girls in the USA [4, 6, 7, 62]. Whereas a major limitation of our study is the small sample size, we show feasibility and provide rich longitudinal data regarding genital immune microenvironment in adolescent girls. This population is difficult to recruit and retain, and therefore under-represented in research and clinical trials, despite bearing a high burden of HIV infections and pregnancies. Another limitation of our data is that the V4 variable region of the 16S rRNA gene has lower resolution for distinguishing between various Lactobacilli species [78, 79]. As a result, we were not able to quantify the relative abundance of all *Lactobacillus* species including *L*. *crispatus* in our samples. This is important because, whereas some *Lactobacilli* species such as *L*. *crispatus* have been associated with reduced risk of HIV/STI acquisition, others such as *L*. *jensenii* were not [80]. At least one study [7] reported lower *L*. *jensenii* in the vagina of adolescents compared to those of adult women. We were also limited by not having access to the data on serum concentration of DMPA, which would be at nadir levels after 3-months at the time of the visit 2 sampling.

Overall, our study fills critical gaps and provides a framework, which can be expanded to larger projects to evaluate associations between STI risk, hormonal contraception and FGT immune milieu along with potential effects of sociodemographic and behavioral factors in adolescent girls.

## Supporting information

S1 Checklist STROBE statement—Checklist of items that should be included in reports of observational studies. (DOCX)

S1 Fig Relative abundance of bacterial taxa. Heatmap showing relative abundances of bacterial taxa identified by 16S rRNA gene sequencing of paired vaginal swab samples from 34 adolescent girls, before and after start of contraception. Rows represent taxa (genus or species) and columns correspond to individual samples. Color indicates relative abundance of the taxon in the sample, from 0 to 1. The three top rows indicate race of participant, visit number, and contraceptive used. The top dendrogram shows three distinct clusters: the left cluster dominated by *Lactobacillus*.*iners*; the center by *Lactobacillus* species other than *iners*; and the right comprising more diverse communities.
(TIF)

S1 Data (XLSX)

S2 Data (CSV)
