# Supplementary material for: Effect of progestin-based contraceptives on HIV-associated vaginal immune biomarkers and microbiome in adolescent girls
Source: PLoS One. 2024 Jul 15;19(7):e0306237. doi: 10.1371/journal.pone.0306237 (PMC11249223; doi:10.1371/journal.pone.0306237)
Supplement: S1 Checklist — (DOCX) [file pone.0306237.s001.docx]

STROBE Statement—checklist of items that should be included in reports of observational studies

|  | Item No. | Recommendation | Page  No. | Relevant text from manuscript |
| --- | --- | --- | --- | --- |
| **Title and abstract** | 1 | (*a*) Indicate the study’s design with a commonly used term in the title or the abstract | 2 | Line 29: The objective of this longitudinal cohort study was to evaluate the effects of short-term use of three progestin-based contraceptives, levonorgestrel intrauterine device (LNG-IUD), subdermal etonogestrel (ETNG), and injectable DMPA, on HIV-associated vaginal immune biomarkers and microbiome in adolescent girls. |
|  |  | (*b*) Provide in the abstract an informative and balanced summary of what was done and what was found | 2 | Lines 32-44: Abstract |
| Introduction | | | |  |
| Background/rationale | 2 | Explain the scientific background and rationale for the investigation being reported | 4-6 | Lines 64-107: Introduction |
| Objectives | 3 | State specific objectives, including any prespecified hypotheses | 5-6 | Lines 104-107: With this in mind, our objective in this study was to longitudinally evaluate short-term effects of three progestin-based contraceptives on vaginal immune biomarkers and microbiome in a cohort of adolescent girls in the Washington DC metro area. |
| Methods | | | |  |
| Study design | 4 | Present key elements of study design early in the paper | 6 | Lines 111-113: This is a longitudinal cohort study, which enrolled sexually active HIV negative adolescent girls aged 15-19 years, from Medstar Washington Hospital Center and the Adolescent Clinic at Children’s National Hospital in Washington DC between 2017-2019. |
| Setting | 5 | Describe the setting, locations, and relevant dates, including periods of recruitment, exposure, follow-up, and data collection | 6 | Lines 111-129: Study design and approval. |
| Participants | 6 | (*a*) *Cohort study*—Give the eligibility criteria, and the sources and methods of selection of participants. Describe methods of follow-up  *Case-control study*—Give the eligibility criteria, and the sources and methods of case ascertainment and control selection. Give the rationale for the choice of cases and controls  *Cross-sectional study*—Give the eligibility criteria, and the sources and methods of selection of participants | 6 | Lines 111-129: Study design and approval. |
|  |  | (*b*) *Cohort study*—For matched studies, give matching criteria and number of exposed and unexposed  *Case-control study*—For matched studies, give matching criteria and the number of controls per case |  | N/A |
| Variables | 7 | Clearly define all outcomes, exposures, predictors, potential confounders, and effect modifiers. Give diagnostic criteria, if applicable | 7-9 | Lines 151-197: Material and Methods: Detection of immune biomarkers, Measurement of anti-HIV activity, Microbiome analysis, Statistical Analysis. |
| Data sources/ measurement | 8* | For each variable of interest, give sources of data and details of methods of assessment (measurement). Describe comparability of assessment methods if there is more than one group | *6-9* | *Lines 111-197: Material and Methods* |
| Bias | 9 | Describe any efforts to address potential sources of bias |  | N/A |
| Study size | 10 | Explain how the study size was arrived at | 9 | Lines 182:186. Power calculation: The study was designed as a pilot, with the aim of recruiting approximately 50 participants, n=10 for each of the 5 groups. This would give us good statistical power only for relatively strong effects. For example, a one-sample t-test would have 80% power to detect a moderately large standardized effect size of 1.0. |

Continued on next page

| Quantitative variables | 11 | Explain how quantitative variables were handled in the analyses. If applicable, describe which groupings were chosen and why | 6-10 | Lines 110-198. Material and Methods |
| --- | --- | --- | --- | --- |
| Statistical methods | 12 | (*a*) Describe all statistical methods, including those used to control for confounding | 9 | Lines 188-198. Material and Methods, Statistical analysis |
|  |  | (*b*) Describe any methods used to examine subgroups and interactions |  | N/A |
|  |  | (*c*) Explain how missing data were addressed | 10 | Lines 194-196. Missing data were minimal (0 for most variables, 1-2 for MIP-1α, MIP-1β, and SLPI measurements, and 5 for SerpinA1). We noted these in table footnotes and excluded cases with missing data from significance tests involving the missing variables. |
|  |  | (*d*) *Cohort study*—If applicable, explain how loss to follow-up was addressed  *Case-control study*—If applicable, explain how matching of cases and controls was addressed  *Cross-sectional study*—If applicable, describe analytical methods taking account of sampling strategy | 10 | Lines 196-198. Data from participants who were lost to follow-up after visit 1 were included in the baseline analyses but excluded from the paired analyses. |
|  |  | (*e*) Describe any sensitivity analyses |  | N/A |
| Results | | | | |
| Participants | 13* | (a) Report numbers of individuals at each stage of study—eg numbers potentially eligible, examined for eligibility, confirmed eligible, included in the study, completing follow-up, and analysed | 9-10  Lines 201-217. Results. Characteristics of study population at baseline and follow-up |  |
|  |  | (b) Give reasons for non-participation at each stage | 10 | Line 209-211. In one case, no sample was collected because the participant was menstruating, and 5 samples were excluded due to presence of blood (2), positive test for Chlamydia (2), or self-treatment with Monostat (1). |
|  |  | (c) Consider use of a flow diagram | N/A |  |
| Descriptive data | 14* | (a) Give characteristics of study participants (eg demographic, clinical, social) and information on exposures and potential confounders | 11 | Table 1 |
|  |  | (b) Indicate number of participants with missing data for each variable of interest | 10 | Lines 192-194. Missing data were minimal (0 for most variables, 1-2 for MIP-1α, MIP-1β, and SLPI measurements, and 5 for SerpinA1). We noted these in table footnotes and excluded cases with missing data from significance tests involving the missing variables. |
|  |  | (c) *Cohort study*—Summarise follow-up time (eg, average and total amount) | 7 | Lines 128-129. One follow-up visit was conducted 3 months after contraceptive initiation and behavioral and biological data was collected same as visit 1. |
| Outcome data | 15* | *Cohort study*—Report numbers of outcome events or summary measures over time | 14, 16 | Table 2. Concentrations of biomarkers at baseline by race and age group.  Table 3: Concentrations of biomarkers at baseline and 3-month follow-up, N = 34 |
|  |  | *Case-control study—*Report numbers in each exposure category, or summary measures of exposure |  |  |
|  |  | *Cross-sectional study—*Report numbers of outcome events or summary measures |  |  |
| Main results | 16 | (*a*) Give unadjusted estimates and, if applicable, confounder-adjusted estimates and their precision (eg, 95% confidence interval). Make clear which confounders were adjusted for and why they were included | 14, 16 | Table 2. Concentrations of biomarkers at baseline by race and age group.  Table 3: Concentrations of biomarkers at baseline and 3-month follow-up, N = 34.  Figures 2 and 3 |
|  |  | (*b*) Report category boundaries when continuous variables were categorized | N/A |  |
|  |  | (*c*) If relevant, consider translating estimates of relative risk into absolute risk for a meaningful time period | N/A |  |

Continued on next page

| Other analyses | 17 | Report other analyses done—eg analyses of subgroups and interactions, and sensitivity analyses | N/A |  |
| --- | --- | --- | --- | --- |
| Discussion | | | | |
| Key results | 18 | Summarise key results with reference to study objectives | 19 | Lines 329-334. Discussion. |
| Limitations | 19 | Discuss limitations of the study, taking into account sources of potential bias or imprecision. Discuss both direction and magnitude of any potential bias | 21 | Lines 374-380. Discussion |
| Interpretation | 20 | Give a cautious overall interpretation of results considering objectives, limitations, multiplicity of analyses, results from similar studies, and other relevant evidence | 21-22 | Lines 370-388. Discussion |
| Generalisability | 21 | Discuss the generalisability (external validity) of the study results | 21-22 | Lines 370-388. Discussion |
| Other information | |  | | |
| Funding | 22 | Give the source of funding and the role of the funders for the present study and, if applicable, for the original study on which the present article is based |  | Submitted online per instructions from journal. |

*Give information separately for cases and controls in case-control studies and, if applicable, for exposed and unexposed groups in cohort and cross-sectional studies.

**Note:** An Explanation and Elaboration article discusses each checklist item and gives methodological background and published examples of transparent reporting. The STROBE checklist is best used in conjunction with this article (freely available on the Web sites of PLoS Medicine at http://www.plosmedicine.org/, Annals of Internal Medicine at http://www.annals.org/, and Epidemiology at http://www.epidem.com/). Information on the STROBE Initiative is available at www.strobe-statement.org.
