# Supplementary figures and images for: Effect of progestin-based contraceptives on HIV-associated vaginal immune biomarkers and microbiome in adolescent girls
Source: PLoS One. 2024 Jul 15;19(7):e0306237. doi: 10.1371/journal.pone.0306237 (PMC11249223; doi:10.1371/journal.pone.0306237)

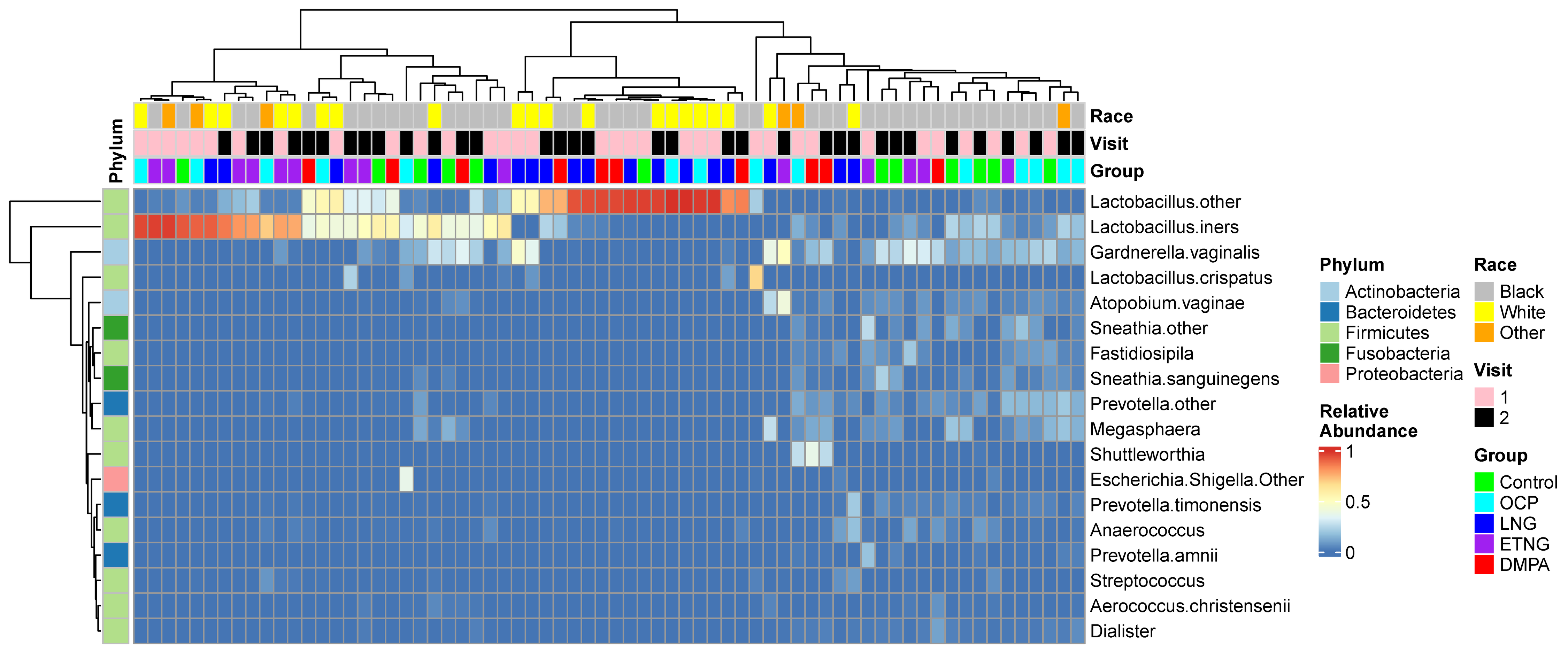

Supplement: S1 Fig — Heatmap showing relative abundances of bacterial taxa identified by 16S rRNA gene sequencing of paired vaginal swab samples from 34 adolescent girls, before and after start of contraception. Rows represent taxa (genus or species) and columns correspond to individual samples. Color indicates relative abundance of the taxon in the sample, from 0 to 1. The three top rows indicate race of participant, visit number, and contraceptive used. The top dendrogram shows three distinct clusters: the left cluster dominated by Lactobacillus.iners; the center by Lactobacillus species other than iners; and the right comprising more diverse communities. (TIF) [file pone.0306237.s002.tif]
